# Tocilizumab in Acute Coronary Syndromes: A Systematic Review of Randomized Controlled Trials

**DOI:** 10.7759/cureus.116359

**Published:** 2026-09-16

**Authors:** Muhammad Umair Iqbal, Hassan Askari, Ira Abid, Bisma Hussain, Muhammad Hanan Abid, Aeyza Nasir

**Affiliations:** 1 Cardiology, Mid Yorkshire Hospitals NHS Trust, Wakefield, GBR; 2 Medicine, King Edward Medical University, Lahore, PAK

**Keywords:** acute coronary syndrome, myocardial infarction, non-st-segment elevation myocardial infarction (nstemi), st-segment elevation myocardial infarction (stemi), tocilizumab

## Abstract

This systematic review synthesizes the randomized controlled trial (RCT) evidence for tocilizumab, an interleukin-6 (IL-6) receptor antagonist, as an adjunctive therapy in acute coronary syndrome (ACS). IL-6 is a central mediator of the inflammatory response that follows myocardial infarction and contributes to ischemia-reperfusion injury, positioning its blockade as a plausible cardioprotective strategy. Following the Preferred Reporting Items for Systematic Reviews and Meta-Analyses (PRISMA) 2020 guidance, we searched PubMed/MEDLINE, Embase, Cochrane Library, and Scopus for RCTs of tocilizumab administered during the acute phase of ST-segment elevation myocardial infarction (STEMI) or non-ST-segment elevation myocardial infarction (NSTEMI) versus placebo or standard of care; risk of bias was assessed using the Cochrane RoB 2.0 tool. Given the substantial heterogeneity in population, dosing, and primary endpoints, findings were synthesized narratively rather than pooled. Four trials comprising 439 analyzed patients met the eligibility criteria, spanning NSTEMI and STEMI populations and both intravenous and subcutaneous dosing regimens, one of which was stopped early for futility. In the three intravenously dosed trials, tocilizumab more than halved the C-reactive protein area under the curve relative to placebo (all p < 0.001), with two trials also showing lower troponin release. One trial additionally demonstrated a significant improvement in myocardial salvage index and reduced microvascular obstruction on cardiac magnetic resonance imaging (CMRI), without significant benefit on the final infarct size or six-month clinical outcomes; a separate, higher-risk trial found no significant benefit on infarct size, troponin, or neurohormonal activation despite comparable suppression of inflammation. Major adverse cardiac events did not differ significantly between arms in either trial assessing this endpoint, and the smallest, subcutaneously dosed trial raised an unreplicated cardiac tamponade signal. Two of the four trials were judged overall low risk of bias. Tocilizumab consistently attenuates the acute inflammatory response and, in one trial, improves imaging surrogates of myocardial salvage, but evidence for clinical benefit is absent, and the trial base remains too small and heterogeneous to support meta-analytic pooling or practice change. Larger, adequately powered trials with harmonized endpoints and blinded safety adjudication are needed.

## Introduction and background

Acute coronary syndrome (ACS), encompassing ST-segment elevation myocardial infarction (STEMI) and non-ST-segment elevation myocardial infarction (NSTEMI), remains a leading cause of morbidity and mortality worldwide despite substantial advances in reperfusion therapy [1,2]. Timely percutaneous coronary intervention (PCI) has markedly improved survival after STEMI, yet a considerable proportion of patients go on to develop heart failure or recurrent ischemic events, and residual risk persists even with optimal revascularization [3]. A growing body of evidence implicates the restoration of blood flow itself as a double-edged intervention: ischemia-reperfusion (I/R) injury, triggered by the abrupt return of oxygenated blood to previously ischemic myocardium, may account for as much as half of the final infarct size in patients treated with primary PCI [4,5]. Inflammatory mechanisms are now recognized as central drivers of this secondary injury, extending damage well beyond that caused by the index ischemic insult [6].

Among the inflammatory mediators implicated in this process, interleukin-6 (IL-6) has attracted particular interest. IL-6 is released early after myocardial injury, contributes to atherosclerotic plaque destabilization, and amplifies the inflammatory cascade that follows reperfusion [7]. Elevated IL-6 levels in the acute phase of myocardial infarction (MI) are associated with larger infarct size, impaired left ventricular function, and worse short- and long-term outcomes. This mechanistic link has positioned IL-6 signaling as a plausible therapeutic target, building on the broader premise established by trials such as CANTOS and the colchicine cardiovascular outcome trials that anti-inflammatory therapy can reduce cardiovascular events in patients with established coronary artery disease [8-10]. Whether this benefit extends to the pharmacologic inhibition of IL-6 specifically, and whether it can be achieved safely in the acute peri-infarction period, has remained comparatively unexplored.

Tocilizumab, a humanized monoclonal antibody that blocks both membrane-bound and soluble IL-6 receptors, is well established in the treatment of rheumatoid arthritis and other autoimmune conditions, with an extensive safety record in those populations [11]. Its repurposing as an adjunct therapy in acute myocardial infarction (AMI) represents a natural extension of the inflammation hypothesis, and over the past decade, a small number of investigator-initiated randomized controlled trials (RCTs) have tested this concept directly. Kleveland et al. first demonstrated that a single infusion of tocilizumab administered prior to coronary angiography reduced the area under the curve (AUC) for both C-reactive protein (CRP) and high-sensitivity troponin T in patients with NSTEMI, with the effect concentrated in patients treated with PCI [12]. Building on this signal, the ASSAIL-MI trial extended the approach to STEMI, showing that tocilizumab administered during primary PCI improved myocardial salvage index on cardiac magnetic resonance imaging (CMRI) and reduced the extent of microvascular obstruction, although the effect on the final infarct size did not reach statistical significance [13]. A third, smaller trial (STAT-MI) attempted to test tocilizumab against a hard clinical endpoint, 30-day major adverse cardiac events (MACE) rather than a surrogate marker, but was stopped early on an interim futility analysis after enrollment stalled, leaving its findings substantially underpowered [14].

Given the small number of available trials, their heterogeneity in population, endpoint, and statistical power, and the presence of an unresolved methodological and safety picture, a formal meta-analytic pooling of effect estimates is neither statistically meaningful nor methodologically defensible at this stage. We therefore conducted a systematic review with narrative synthesis to comprehensively summarize the existing RCT evidence on tocilizumab in ACS, spanning both STEMI and NSTEMI populations. Our objectives were to characterize the consistency and clinical significance of tocilizumab's effects across inflammatory, imaging, and clinical outcome domains; to critically appraise the methodological quality and risk of bias of the available trials; and to identify the evidence gaps that must be addressed before tocilizumab can be considered for larger, adequately powered clinical outcome trials in this population.

## Review

Materials and methods

This systematic review was conducted in accordance with the Preferred Reporting Items for Systematic Reviews and Meta-Analyses (PRISMA) 2020 statement. This review was not registered on the International Prospective Register of Systematic Reviews (PROSPERO) or an equivalent registry.

Eligibility Criteria

Studies were eligible if they met the following PICOS criteria: (1) for population, adult patients (≥18 years) presenting with ACS, defined as STEMI or NSTEMI; (2) for intervention, tocilizumab administered during the acute phase of presentation, by any dose or route; (3) for comparator, placebo or standard of care alone; (4) for outcome, inflammatory biomarker kinetics (CRP, troponin), CMRI-derived measures (myocardial salvage index, infarct size, microvascular obstruction), MACE, mortality, or safety/adverse events; and (5) for study design, RCTs only. We excluded observational cohort and case-control studies of tocilizumab given the absence of comparable real-world exposure data in this specific indication (tocilizumab is not standard of care for ACS outside trial settings), mechanistic or biomarker sub-studies derived from an already-included parent trial cohort, studies of tocilizumab in non-ACS cardiac populations (e.g., post-cardiac arrest, chronic heart failure), and studies evaluating chronic tocilizumab exposure in unrelated indications (e.g., rheumatoid arthritis cardiovascular safety cohorts) where MI was assessed as an incident safety outcome rather than as the primary treatment target. No language or publication date restrictions were applied (Appendix A).

Search Strategy

We searched PubMed/MEDLINE, Embase, Cochrane Library, and Scopus from database inception to April 30, 2026. The search strategy combined controlled vocabulary and free-text terms for "tocilizumab" OR "IL-6 receptor antagonist" OR "interleukin-6 receptor inhibitor" AND "myocardial infarction" OR "acute coronary syndrome" OR "STEMI" OR "NSTEMI". Reference lists of all included trials and of relevant prior reviews on IL-6/IL-1β/NLRP3 pathway inhibition in coronary artery disease were hand-searched to identify additional eligible studies. Ongoing and unpublished trials were identified through ClinicalTrials.gov to inform the discussion of the current evidence gap (Appendix B).

Study Selection and Data Extractions

Two reviewers (B.H. and M.H.A.) independently screened titles/abstracts and then full texts against the eligibility criteria, with disagreements resolved by discussion or a third reviewer (H.A.). Data were extracted independently in duplicate using a piloted, standardized extraction form covering trial identifiers (registry number, publication), design and setting, population characteristics (ACS subtype, baseline demographics, and risk scores), intervention details (dose, route, timing relative to symptom onset and revascularization), comparator, pre-specified primary and secondary outcomes with point estimates and measures of dispersion, and safety/adverse event data. Where a trial's registry entry, protocol paper, and primary results paper reported overlapping information, the primary results publication was treated as the source of record. Corresponding authors were not contacted for unreported data given the small number of eligible trials and the completeness of publicly available reporting.

Risk of Bias

Risk of bias in the included trials was assessed independently by two reviewers (A.N. and I.A.) using the Cochrane Risk of Bias 2.0 (RoB 2.0) tool across five domains, namely, randomization process, deviations from intended interventions, missing outcome data, measurement of the outcome, and selection of the reported result, each rated as low risk, some concerns, or high risk, with an overall judgment derived per Cochrane algorithm. Discrepancies were resolved by consensus.

Data Synthesis

Given that only four RCTs met the inclusion criteria, with heterogeneous ACS populations (STEMI-only, NSTEMI-only, and mixed/unstratified), differing primary endpoints (biomarker AUC, CMRI-based myocardial salvage, and 30-day MACE, respectively), and substantially different sample sizes, we determined that formal meta-analysis was not appropriate for the majority of outcomes. We planned quantitative pooling only where two or more trials reported a conceptually equivalent outcome using comparable timepoints and metrics; all other outcomes were synthesized narratively, grouped by outcome domain (inflammatory biomarkers, imaging/infarct-related outcomes, clinical events, and safety). Where meaningful, subgroup and sensitivity considerations reported within individual trials (e.g., stratification by revascularization status or time from symptom onset) were incorporated into the narrative rather than treated as independent evidence.

Results

Study Selection

A systematic search of PubMed, Cochrane Library, Scopus, and Embase yielded 2,059 records. Following deduplication (n = 929 removed), 1,130 titles and abstracts were screened in accordance with the pre-specified inclusion and exclusion criteria. Nine studies proceeded to full-text review, of which four RCTs met the final eligibility criteria and were included in the qualitative synthesis. The selection process is presented in the PRISMA 2020 flow diagram (Figure 1).

**Figure 1:**
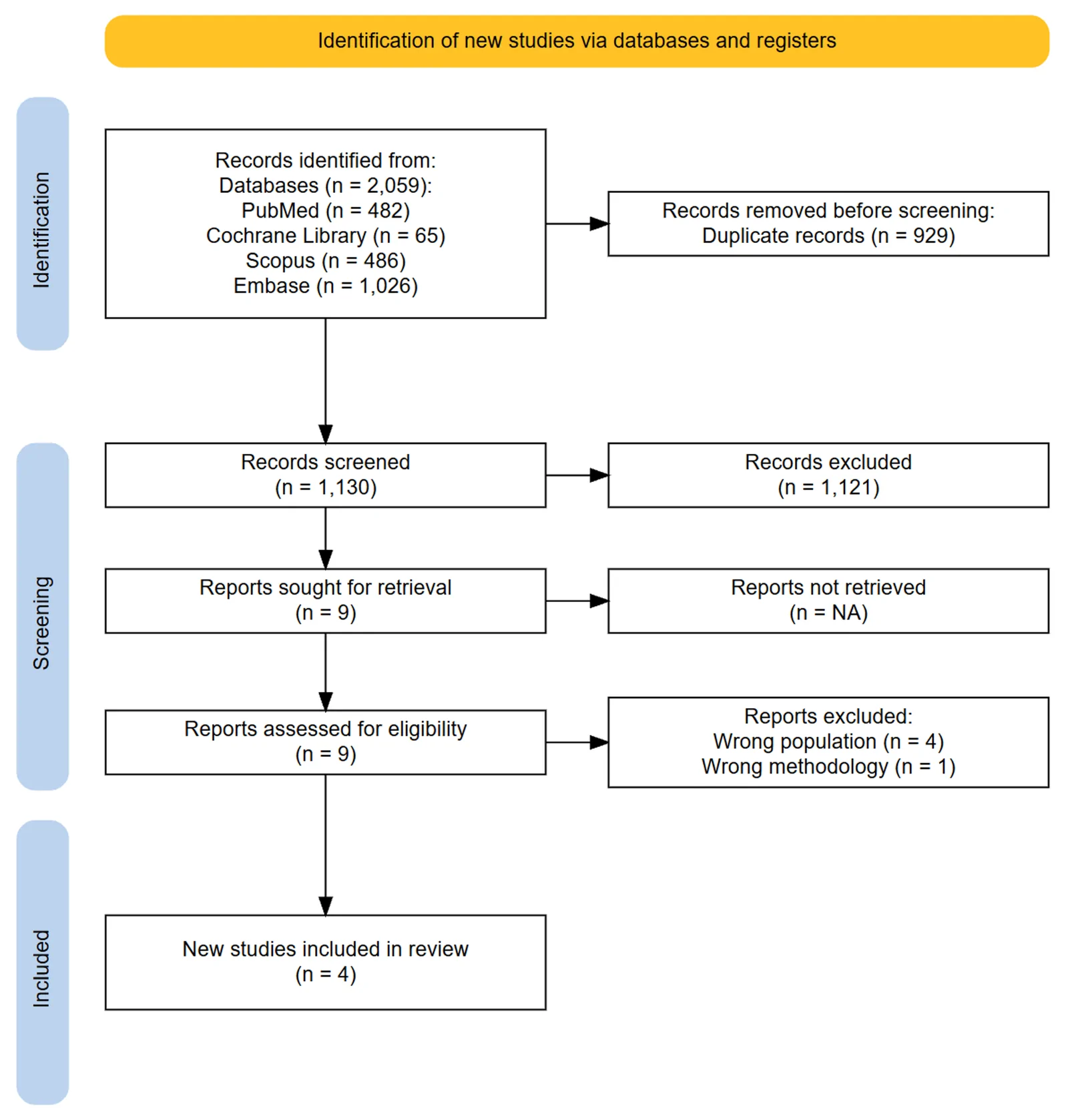
PRISMA diagram illustrating the study selection process. PRISMA: Preferred Reporting Items for Systematic Reviews and Meta-Analyses

Study Characteristics

The four included trials analyzed a combined 439 patients with ACS (Table 1). Population and design varied by trial: Kleveland et al. enrolled 121 patients (117 analyzed) with NSTEMI at two Norwegian centers, randomized at a median of two days after symptom onset to a single intravenous 280 mg dose of tocilizumab or placebo prior to coronary angiography [12]. Broch et al. randomized 199 patients with STEMI at three Norwegian centers, presenting within six hours of symptom onset, to the same 280 mg intravenous dose of tocilizumab or matching placebo, given during primary PCI [13]. Kunkel et al. randomized 100 patients (49 tocilizumab/51 placebo) with AMI (73% anterior STEMI) to the same 280 mg intravenous dose given rapidly after PCI within a 2 × 2 factorial design that additionally randomized patients to low-dose dobutamine or placebo [15]. Carroll et al. enrolled 28 patients (of a planned 125) with either STEMI or NSTEMI at a single US center, randomized to a lower, subcutaneous 162 mg dose given within 24 hours of admission; this trial was stopped early following an unplanned interim futility analysis [14].

**Table 1:** Study characteristics of the included studies. ACS: acute coronary syndrome; PCI: percutaneous coronary intervention; TCZ: tocilizumab; NSTEMI: non-ST-segment elevation myocardial infarction; STEMI: ST-segment elevation myocardial infarction; IQR: interquartile range; AMI: acute myocardial infarction; ORBI: Observatoire Régional Breton sur l'Infarctus risk score; CMRI: cardiac magnetic resonance imaging

Study	Design	Setting	Population (ACS type)	N randomized (analyzed)	Intervention vs. comparator
Kleveland et al. (2016) [12]	Two-center, phase II, double-blind, randomized, placebo-controlled trial	Oslo University Hospital, Rikshospitalet, and St. Olav's Hospital, Trondheim, Norway	NSTEMI, age 18-80, scheduled for coronary angiography; randomized at a median of 2 days (IQR 1-3) after symptom onset	121 enrolled; 117 analyzed (59 placebo/58 tocilizumab)	Single IV infusion tocilizumab 280 mg vs. matching placebo (0.9% NaCl), given prior to coronary angiography
Broch et al. (2021) [13]	Three-center, phase II, parallel-arm, double-blind, randomized, placebo-controlled trial	Oslo University Hospital, Rikshospitalet, Oslo University Hospital, Ullevål, and St. Olav's Hospital, Trondheim, Norway	STEMI, age 18-80, presenting within 6 hours of symptom onset	200 enrolled; 1 withdrew consent, leaving 199 randomized (101 tocilizumab/98 placebo); 4 excluded from primary endpoint analysis due to missing CMRI, leaving 195 analyzed for primary endpoint (99 tocilizumab/96 placebo)	Single IV infusion tocilizumab 280 mg over 1 hour vs. matching placebo, given during PCI
Kunkel et al. (2026) [15]	Single-center, phase II, randomized, double-blind, placebo-controlled trial	Copenhagen University Hospital, Rigshospitalet, Denmark	AMI (73% anterior STEMI) without cardiogenic shock at hospital admission, at intermediate-to-high risk of cardiogenic shock development (ORBI score ≥10), treated with PCI <24 hours from symptom onset	100 randomized (49 tocilizumab/51 placebo)	Single IV infusion tocilizumab 280 mg over 1 hour vs. matching placebo, given rapidly after PCI; co-randomized 1:1 to 24-hour low-dose dobutamine or placebo (tocilizumab effect estimates adjusted for this allocation)
Carroll et al. (2018) [14]	Single-center, randomized, double-blind, placebo-controlled, non-inferiority design; stopped early for futility	Keesler AFB Medical Center, Biloxi, Mississippi, USA	AMI (STEMI + NSTEMI, not stratified), age ≥18	28 enrolled (12 tocilizumab/16 placebo); 11 tocilizumab analyzed for primary outcome (1 lost to follow-up)	Single subcutaneous injection tocilizumab 162 mg vs. saline placebo, within 24 hours of admission

Baseline characteristics were broadly balanced between treatment arms within each trial (Table 2). Patients in Carroll et al. were older (67.7-70.7 years) than those in the two Norwegian trials (59.8-62 years) and had a substantially higher prevalence of hypertension (83-94% vs. 29-45%) and diabetes (63-67% vs. 17-19%), consistent with its unselected, real-world enrollment relative to the stricter eligibility criteria of Kleveland et al. and Broch et al. (both of which excluded patients with prior MI, among other criteria) [12-14]. Kunkel et al.'s population, selected for elevated cardiogenic shock risk, showed a similar pattern of higher comorbidity burden in the tocilizumab arm specifically, with numerically higher hypertension (67% vs. 43%) and diabetes (33% vs. 8%) than placebo, an imbalance the trial's own investigators noted and adjusted for statistically [15]. PCI was performed in the majority of patients across all four trials (67-100%).

**Table 2:** Baseline characteristics by treatment arm. Values are presented as mean ± SD or n (%) unless otherwise stated. *Kleveland et al. [12] reports maximum TnT before baseline as a geometric mean (95% CI) across n = 85 with available data, not the full cohort. TCZ: tocilizumab; BMI: body mass index; TnT: troponin T; TnI: troponin I; MI: myocardial infarction; GRACE: Global Registry of Acute Coronary Events; PCI: percutaneous coronary intervention; IQR: interquartile range; NR: not reported

Characteristic	Kleveland et al. (2016) [12]		Broch et al. (2021) [13]		Kunkel et al. (2026) [15]		Carroll et al. (2018) [14]	
	Placebo (n = 59)	TCZ (n = 58)	Placebo (n = 98)	TCZ (n = 101)	Placebo (n = 49)	TCZ (n = 51)	Placebo (n = 16)	TCZ (n = 12)
Age, years	60.1 ± 9.9	59.8 ± 7.7	60 ± 9	62 ± 10	NR (overall median 68y)	NR (overall median 68y)	67.7 ± 9.5	70.7 ± 10.0
Female sex	5 (8.5%)	9 (15.5%)	11 (11%)	21 (21%)	NR	NR	2 (12.5%)	2 (16.7%)
BMI, kg/m²	27.4 ± 4.4	28.8 ± 3.3	27.5 ± 4.3	27.1 ± 4.5	NR	NR	29.4 ± 3.5	28.4 ± 4.0
Hypertension	17 (28.8%)	26 (44.8%)	30 (31%)	33 (33%)	22 (43%)	33 (67%)	15 (93.8%)	10 (83.3%)
Diabetes mellitus	10 (16.9%)	11 (19%)	6 (6%)	8 (8%)	4 (8%)	16 (33%)	10 (62.5%)	8 (66.7%)
Current smoking	17 (28.8%)	15 (26.3%)	38 (39%)	30 (30%)	NR	NR	NR	NR
Prior MI	7 (11.9%)	9 (15.5%)	Excluded (prior MI was an exclusion criterion)	Excluded (prior MI was an exclusion criterion)	NR	NR	5 (31.3%)	7 (58.3%)
GRACE risk score	90.3 ± 20.2	86.3 ± 19.1	135 ± 21	140 ± 25	NR	NR	NR	NR
Baseline TnT (diagnostic sample)	TnT 310 ng/L* (geometric mean)	TnT 334 ng/L* (geometric mean)	TnT 49 (28-95) ng/L (median, IQR)	TnT 44 (22-163) ng/L (median, IQR)	TnT 1,550 ng/L	TnT 1,060 ng/L	Peak TnI (6.97 ± 9.64) ng/mL	Peak TnI (23.82 ± 56.22) ng/mL
PCI performed	47/59 (79.7%)	41/58 (70.7%)	All underwent coronary angiography	All underwent coronary angiography	100%	100%	12 (75%)	8 (66.6%)
Time, symptom onset to treatment	Median 2 days (IQR 1-3)	Median 2 days (IQR 1-3.5)	151 ± 71 min (to PCI center arrival)	149 ± 72 min (to PCI center arrival)	NR	NR	Within 24 hours of admission (protocol-defined)	Within 24 hours of admission (protocol-defined)

Inflammatory Biomarkers

CRP kinetics were the most consistently reported outcome across all four trials, although timepoints and metrics differed (Table 3). In Kleveland et al., the pre-specified primary endpoint, AUC for high-sensitivity CRP during hospitalization, was significantly lower with tocilizumab than placebo (2.0 vs. 4.2 mg/L/hours; placebo 2.1-fold higher; p < 0.001) [12]. Broch et al. similarly found a substantially lower CRP AUC with tocilizumab (1.9 vs. 8.6 mg/L/hours; p < 0.001) [13]. Kunkel et al. reported a comparable pattern of sustained suppression, with a peak CRP of 78% lower with tocilizumab than placebo across all measured timepoints (ratio 0.22; 95% CI: 0.15-0.32; p < 0.001) [15]. In contrast, Carroll et al. observed a numerical increase in CRP from pre- to post-treatment in the tocilizumab arm (20.9 → 57.3 mg/L) that was proportionally larger than the increase observed with placebo (6.0 → 14.4 mg/L), though this difference did not reach statistical significance; the authors attributed this unexpected direction to the lower subcutaneous dose, delayed absorption kinetics, and/or background inflammation from procedural interventions rather than a true paradoxical drug effect [14]. Troponin release followed a broadly concordant pattern in the three adequately dosed, intravenous trials: Kleveland et al. reported a significantly lower high-sensitivity troponin T AUC with tocilizumab (159 vs. 234 ng/L/hours; placebo 1.5-fold higher; p = 0.007), while Broch et al. observed a numerically lower troponin T AUC that did not reach statistical significance (1,614 vs. 2,357 ng/L/hours; p = 0.13), and Kunkel et al. found no difference in peak troponin T between arms (3,579 vs. 3,363 ng/L; p = 0.77) [12,13,15].

**Table 3:** Primary and secondary efficacy outcomes. Values are presented as mean ± SD, median (IQR/range), or n (%) as reported in each source. STAT-MI's MACE definition included recurrent MI, new arrhythmia, septal/valve rupture, dissection, pericarditis, or tamponade; some patients experienced more than one component event, so component counts do not necessarily sum to the composite total per arm as tabulated in the source. Carroll et al. [14] was stopped early on interim futility analysis (n = 28 of a planned 125), so its point estimates carry substantial imprecision. AUC: area under the curve; CMRI: cardiac magnetic resonance imaging; LVEDV: left ventricular end-diastolic volume; MACE: major adverse cardiac events; CRP: C-reactive protein; hsTnT: high-sensitivity troponin T; NT-proBNP: N-terminal pro-B-type natriuretic peptide; MI: myocardial infarction; IQR: interquartile range; NS: not significant

Study	Outcome	Tocilizumab	Placebo	Effect estimate/p-value
Kleveland et al. (2016) [12]	Primary: CRP AUC during hospitalization (days 1-3)	2.0 mg/L/hours (median)	4.2 mg/L/hours (median)	Placebo 2.1× higher; p < 0.001
	Key secondary: hsTnT AUC during hospitalization	159 ng/L/hours (median)	234 ng/L/hours (median)	Placebo 1.5× higher; p = 0.007
	MACE during the 6-month follow-up	3 events	6 events	No significant between-group difference
Broch et al. (2021) [13]	Primary: myocardial salvage index, % (CMRI, days 3-7)	69.3 ± 19.3 (n = 99)	63.6 ± 20.8 (n = 96)	Δ 5.6 (95% CI: 0.2-11.3); p = 0.04
	Final infarct size at 6 months, % LV mass	7.2 (2.6-11.8) (n = 97)	9.1 (2.9-16.3) (n = 93)	p = 0.08 (NS)
	Microvascular obstruction, % LV volume	0 (0-14) (n = 99)	4 (0-18) (n = 96)	p = 0.03
	Troponin T AUC, ng/L/hours	1,614 (860-3,515) (n = 101)	2,357 (97-4,127) (n = 98)	p = 0.13 (NS)
	CRP AUC, mg/L/hours	1.9 (0.9-4.9) (n = 101)	8.6 (5.0-17.9) (n = 98)	p < 0.001
	LVEDV at 6 months, mL (baseline-adjusted)	157 (151-166) (n = 97)	160 (153-166) (n = 93)	Δ 3 (95% CI: −6 to 11); p = 0.54 (NS)
	NT-proBNP at 6 months, ng/L	79 (50-187) (n = 98)	63 (50-148) (n = 97)	Δ 32 (95% CI: −84 to 149); p = 0.25 (NS)
Kunkel et al. (2026) [15]	Primary: peak NT-proBNP ≤48 hours	3,355 ng/L (mean, n = 49)	4,002 ng/L (mean, n = 51)	Ratio 0.82 (95% CI: 0.62-1.10); p = 0.18
	Secondary: NT-proBNP at 12 hours	-	-	Ratio 0.78 (95% CI: 0.61-0.99); p=0.04
	Secondary: peak CRP	-	-	Ratio 0.22 (95% CI: 0.15-0.32); p<0.001
	Secondary: peak troponin T, ng/L	3,579 (median)	3,363 (median)	Ratio 1.06 (95% CI: 0.70-1.62); p=0.77
	Secondary: acute infarct size, 48 hours, % LV mass	24% (n = 36)	23% (n = 45)	Δ 1% (95% CI: −6 to 8); p = 0.76
	Secondary: final infarct size, 3 months, % LV mass	24% (n = 31)	22% (n = 39)	Δ 2% (95% CI: −5 to 9); p = 0.57
	Death (component of composite safety endpoint)	2/49 (4%)	1/51 (2%)	Not independently powered
Carroll et al. (2018) [14]	Primary: MACE at 30 days (composite, any component)	9 events (11 patients)	3 events (16 patients)	No statistically significant difference reported by trial investigators (cumulative component events, not unique patients; underpowered; stopped for futility)
	Recurrent MI	2/11 (18.2%)	1/16 (6.3%)	-
	Dysrhythmia	2/11 (18.2%)	1/16 (6.3%)	-
	Septal/valve rupture	1/11 (9.1%)	1/16 (9.1%)	-
	Pericarditis	2/11 (18.2%)	0/16 (0%)	-
	Cardiac tamponade	2/11 (18.2%)	0/16 (0%)	Adjudicated as secondary to MI/procedural complication
	Death (30 days)	0/11 (0%)	0/16 (0%)	-
	CRP, mg/L (pre- → post-treatment)	20.9 ± 20.0 → 57.3 ± 89.6	6.0 ± 9.2 → 14.4 ± 30.2	Increase in tocilizumab arm, not statistically significant

Infarct-Related Outcomes

CMRI-based outcomes were reported by two trials, with differing findings [13,15]. The pre-specified primary endpoint of Broch et al., myocardial salvage index at 3-7 days, was significantly higher with tocilizumab than placebo (69.3 ± 19.3% vs. 63.6 ± 20.8%; between-group difference 5.6 percentage points; 95% CI: 0.2-11.3; p = 0.04) [13]. Microvascular obstruction was significantly less extensive with tocilizumab (median 0% vs. 4% of left ventricular volume; p = 0.03). However, the final infarct size at six months (7.2% vs. 9.1% of left ventricular mass; p = 0.08), baseline-adjusted left ventricular end-diastolic volume (LVEDV) at six months (157 vs. 160 mL; p = 0.54), and N-terminal pro-B-type natriuretic peptide (NT-proBNP) at six months (79 vs. 63 ng/L; p = 0.25) did not differ significantly between arms. In contrast, Kunkel et al., studying a higher-risk population selected for elevated cardiogenic shock risk, found no difference in CMRI-quantified infarct size between tocilizumab and placebo at either 48 hours (24% vs. 23% of left ventricular mass; p = 0.76) or three months (24% vs. 22%; p = 0.57) [15]. Infarct sizes in this trial (23-24% of left ventricular mass) were substantially larger than in Broch et al. (7-9%), reflecting the higher-risk, shock-prone population selected by the Observatoire Régional Breton sur l'Infarctus risk score (ORBI) enrollment criterion [15]. Kunkel et al. additionally assessed peak NT-proBNP within 48 hours as their primary endpoint, a neurohormonal marker not reported by the other three trials at this early timepoint; this did not differ significantly between arms over the full 48-hour window (ratio 0.82; 95% CI: 0.62-1.10; p = 0.18), though a significant 22% reduction was observed at the 12-hour timepoint (ratio 0.78; p = 0.04) that was not sustained [15].

Clinical Events

MACE was assessed as a formal endpoint only in Kleveland et al. (as a secondary, exploratory outcome; three events with tocilizumab vs. six with placebo over six months) and Carroll et al. (as the primary outcome) [12,14]. In Carroll et al., MACE events were more numerous with tocilizumab than placebo (nine vs. three events among 11 and 16 patients analyzed, respectively), driven by recurrent MI (two vs. one), dysrhythmia (two vs. one), pericarditis (two vs. zero), and cardiac tamponade (two vs. zero), with overlapping events in at least two tocilizumab-treated patients; the trial investigators reported no statistically significant between-group difference, and the trial was substantially underpowered following early termination [14]. Kunkel et al. recorded death as a component of their composite safety endpoint (two deaths with tocilizumab vs. one with placebo), the only deaths reported across the four included trials [15].

Safety Profile

Overall serious adverse event (SAE) rates did not differ significantly between arms in Kleveland et al. (16 SAEs in 12 tocilizumab-treated patients vs. 27 SAEs in 17 placebo-treated patients), Broch et al. (19 vs. 15 SAEs; p = 0.57), or Kunkel et al., and no myocardial ruptures occurred in either arm of the latter (Table 4) [12,13,15]. Recurrent MI as an adverse event was numerically less frequent with tocilizumab in Broch et al. (zero vs. four events) but more frequent in Carroll et al. (two vs. one event) [13,14]. The most notable safety signal was the occurrence of cardiac tamponade in two of 11 evaluable tocilizumab-treated patients (18.2%) in Carroll et al. versus none in the placebo arm [14]. These events are thought to be secondary to the index MI and a procedural complication rather than directly attributable to tocilizumab, though this adjudication was post hoc and unblinded. No corresponding tamponade events were reported in Kleveland et al., Kunkel et al., or Broch et al., each of which used a higher intravenous dose [12,13,15].

**Table 4:** Safety and adverse events. Values are presented as n or n/N (%) as reported. SAE: serious adverse event

Study	Safety outcome	Tocilizumab	Placebo	Notes
Kleveland et al. (2016) [12]	SAEs (6-month follow-up)	16 SAE in 12 patients	27 SAE in 17 patients	No significant between-group difference
	No safety issues detected over 6 months	-	-	Investigator statement
Broch et al. (2021) [13]	Any SAE	19	15	p = 0.57
	Infections requiring hospitalization	3	2	-
	New malignancy	2	0	-
	Cardiovascular events (total)	9	10	-
	Recurrent MI	0	4	-
	Myocardial rupture	0	0	None in either arm
	Death (6 months)	0	0	-
Kunkel et al. (2026) [15]	Composite safety endpoint (in-hospital)	7% (n = 49)	14% (n = 51)	p = 0.32
	Composite safety endpoint (3 months)	13% (n = 49)	22% (n = 51)	p = 0.3
	Death	2	1	-
	Serious infection	1	6	-
Carroll et al. (2018) [14]	Leukopenia	0/11	0/16	-
	Infection	0/11	0/16	-
	Symptomatic bradycardia	0/11	1/16	-
	Symptomatic hypotension	0/11	1/16	-
	Gross hematuria	1/11	0/16	-
	Re-hospitalization (30 days)	3/11	1/16	-

Risk of Bias

Risk of bias across the four included trials was assessed using the Cochrane RoB 2.0 tool (Figure 2). Kleveland et al. and Broch et al. were judged overall low risk of bias across all five domains, reflecting their robust randomization procedures, matched double-blind placebo, complete or near-complete outcome ascertainment, objective outcome measurement (biochemical assays and core-laboratory-read CMRI, respectively), and pre-specified, fully reported primary and secondary endpoints [12,13]. Carroll et al. was judged low risk across the first four domains (randomization process, deviations from intended interventions, missing outcome data, and measurement of the outcome) but raised some concerns in the domain of selection of the reported result, driven primarily by its early termination following an unplanned interim futility analysis and the absence of a fully pre-specified analysis plan for its composite MACE outcome, factors that can influence which results are ultimately reported and interpreted [14]. Kunkel et al. was judged low risk of bias across four of the five domains but raised some concerns in the domain of missing outcome data, driven by incomplete CMRI follow-up (81% at 48 hours, 70% at three months) for the infarct-size secondary endpoint, a limitation the trial's own investigators attributed to the challenges of imaging a hemodynamically at-risk population [15]. No trial was judged high risk of bias in any domain.

**Figure 2:**
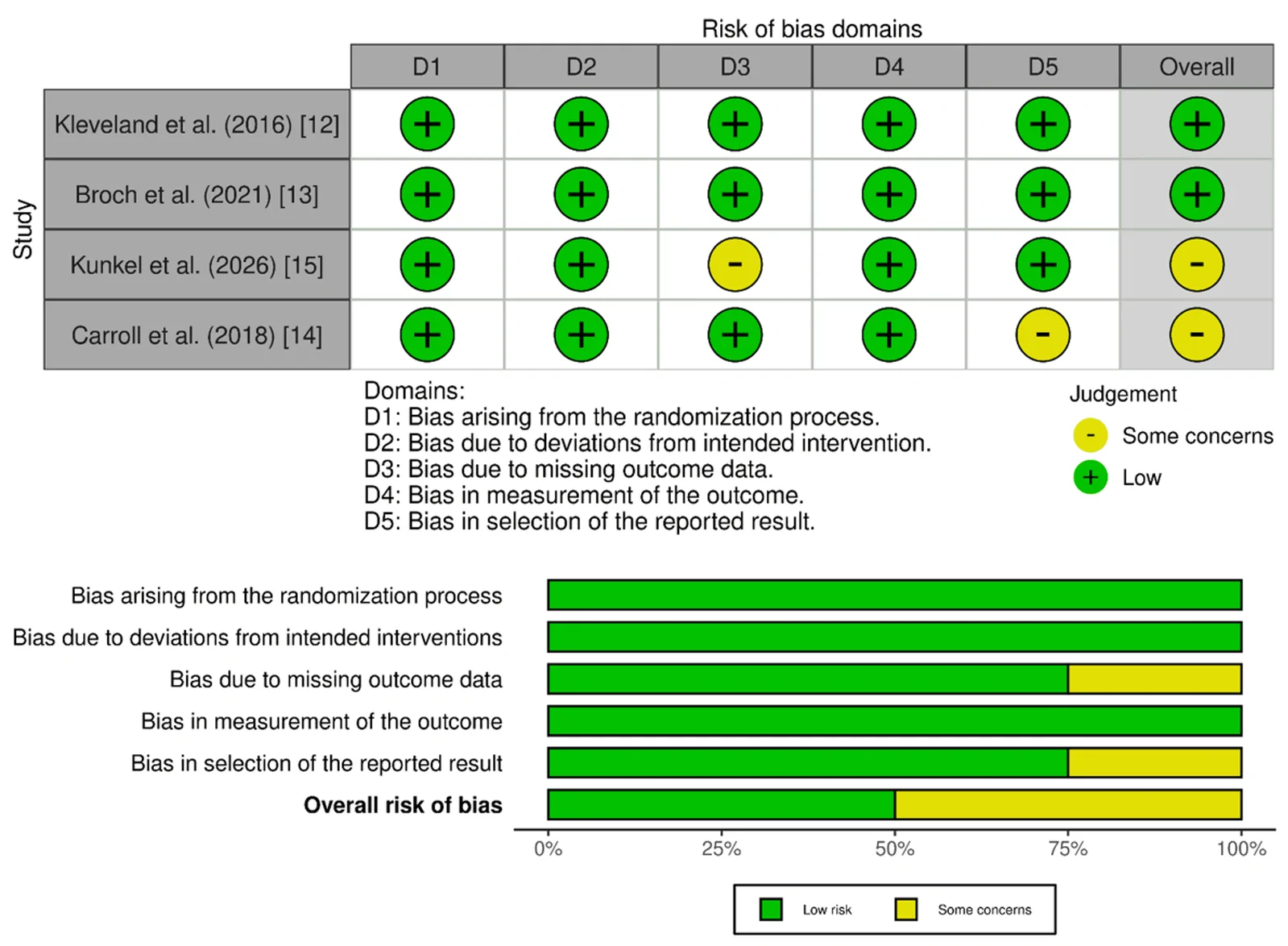
Traffic light plot and summary plot for risk of bias assessment.

Discussion

This systematic review synthesizes the entire published RCT evidence base, comprising four trials and 439 analyzed patients, for tocilizumab as an adjunctive therapy in acute STEMI and NSTEMI. Across the three adequately dosed, intravenously administered trials, tocilizumab consistently and substantially attenuated the acute inflammatory response, more than halving the AUC for CRP in all three studies, with a less consistent reduction in troponin release [12,13,15]. In Broch et al., this anti-inflammatory effect translated into a significant, albeit modest, improvement in myocardial salvage index and a reduction in microvascular obstruction, without a corresponding significant reduction in the final infarct size or six-month clinical and structural outcomes [13]. In Kunkel et al., which enrolled a higher-risk population selected for elevated cardiogenic shock risk, comparable suppression of inflammation did not translate into any measurable benefit on infarct size, troponin release, or neurohormonal activation, suggesting that the anti-inflammatory effect of tocilizumab does not reliably extend to structural or biomarker-based measures of myocardial injury in this population [15]. The smaller, subcutaneously dosed trial demonstrated neither a CRP benefit nor a reduction in MACE and additionally raised a cardiac tamponade signal in two of 11 tocilizumab-treated patients that none of the three larger, intravenously dosed trials reproduced or were able to refute, given the differences in dose, route of administration, and event ascertainment between studies [14].

This pattern, in which a clear proximal biomarker and imaging signal is not accompanied by a corresponding distal clinical signal, mirrors the trajectory observed with other targeted anti-inflammatory strategies in coronary artery disease. The CANTOS trial demonstrated that interleukin-1β inhibition reduces cardiovascular events only following prolonged exposure in a secondary prevention population, whereas smaller acute-phase anti-inflammatory trials, including those evaluating anakinra and methotrexate in STEMI, have shown biomarker or left ventricular remodeling benefit without consistent benefit on hard clinical outcomes at the scale so far studied [16]. The evidence for tocilizumab in ACS remains at this earlier stage: proof of mechanism has been established, but the available trials are neither individually nor collectively powered to detect effects on MACE, mortality, or heart failure hospitalization.

The cardiac tamponade signal observed in Carroll et al. warrants particular caution rather than dismissal [14]. Although these events were adjudicated post hoc as secondary to the index infarction and associated procedural complications, this adjudication was performed without blinding, and the small trial size means the true underlying event rate could plausibly range from negligible to clinically significant. The remaining three trials reported no pericardial complications, although they employed a higher intravenous dose and more restrictive exclusion criteria, so this discrepancy cannot be confidently attributed to a true dose-dependent difference in risk rather than a small trial artifact.

This review is subject to several limitations inherited directly from the underlying evidence base rather than from the conduct of the review itself. Only four trials met the eligibility criteria, which precluded meaningful quantitative pooling for any outcome. Populations, dosing regimens, routes of administration, and primary endpoints differed substantially across the four included trials, ranging from a biomarker AUC to a neurohormonal marker to a CMRI-derived index to a composite clinical endpoint, and this heterogeneity is itself an important finding of the review rather than merely a limitation to be noted in passing. We therefore elected to conduct a narrative synthesis rather than a forced meta-analysis, in order to avoid conveying a degree of statistical precision that this evidence base cannot genuinely support. The early termination of STAT-MI following an unplanned futility analysis, its unmatched placebo formulation, and its reliance on unblended post hoc adjudication of clinical events further limit the confidence that can be placed in its clinical event and safety findings specifically, independent of the broader cross-trial heterogeneity already described [14]. Kunkel et al.'s 2×2 factorial design, in which patients were co-randomized to dobutamine or placebo, introduces an additional layer of complexity; although the trial's own analyses adjusted tocilizumab effect estimates for this allocation, the possibility of an interaction between the two interventions cannot be entirely excluded [15]. Additionally, our review restricted eligibility to RCTs of tocilizumab administered during the acute treatment phase, and while we deliberately excluded observational cohorts of chronic tocilizumab exposure in rheumatoid arthritis populations on methodological grounds, this means our safety conclusions cannot draw on the considerably larger, if indirectly applicable, chronic-exposure safety literature for this agent.

Future trials in this area should prioritize standardized, adequately powered designs with harmonized clinical endpoints applied consistently across STEMI and NSTEMI populations, rather than the mixed or endpoint-inconsistent designs seen to date. Prospective and blinded adjudication of safety events, ideally by an independent clinical events committee, should be regarded as a minimum methodological standard for future work in this area. Dose-ranging or dose-comparison studies may also be warranted to clarify whether the divergent findings between the higher-dose intravenous trials and the lower-dose subcutaneous STAT-MI trial reflect a genuine dose-response relationship for both efficacy and safety.

## Conclusions

The entire published RCT evidence base for tocilizumab in ACS, four trials and 439 analyzed patients, demonstrates a consistent proximal anti-inflammatory effect but no reproducible benefit on the final infarct size, neurohormonal activation, ventricular remodeling, or clinical events; a modest improvement in myocardial salvage and microvascular obstruction on CMRI was observed in one trial but not replicated in a second, higher-risk trial that also assessed infarct size. No trial has been adequately powered to detect differences in MACE or mortality. A tamponade signal in the smallest trial remains unresolved and warrants dedicated safety monitoring in future studies. Given the limited number of trials, their substantial methodological heterogeneity, and this unresolved safety question, current evidence supports proof of mechanism for IL-6 blockade in AMI but not meta-analytic pooling or clinical practice change.

## Appendices

Appendix A

**Table 5:** Population, Intervention, Control, Outcomes, and Study design (PICOS) framework.

Component	Criteria
Population	Adult patients (≥18 years) presenting with acute coronary syndrome (ACS), defined as ST-segment elevation myocardial infarction (STEMI) or non-ST-segment elevation myocardial infarction (NSTEMI)
Intervention	Tocilizumab administered during the acute phase of presentation, by any dose or route
Comparator	Placebo or standard of care alone
Outcome	Inflammatory biomarker kinetics (C-reactive protein, troponin); cardiac magnetic resonance imaging-derived measures (myocardial salvage index, infarct size, microvascular obstruction); major adverse cardiac events (MACE); mortality; safety/adverse events
Study design	Randomized controlled trials only

Appendix B

**Table 6:** Search strategy and results for each database search.

Database	Search string	Results
PubMed	("tocilizumab"[Supplementary Concept] OR "tocilizumab"[All Fields] OR ("interleukin-6"[All Fields] AND ("antagonist*"[All Fields] OR "inhibitor*"[All Fields] OR "receptor blockade"[All Fields] OR "receptor inhibition"[All Fields])) OR ("IL-6 receptor antagonist"[All Fields] OR "IL-6R antagonist"[All Fields] OR "interleukin-6 receptor inhibitor"[All Fields] OR "anti-IL-6"[All Fields])) AND ("myocardial infarction"[MeSH Terms] OR "acute coronary syndrome"[MeSH Terms] OR ("myocardial infarction"[All Fields] OR "acute coronary syndrome"[All Fields] OR ("ST elevation myocardial infarction"[MeSH Terms] OR ("st"[All Fields] AND "elevation"[All Fields] AND "myocardial"[All Fields] AND "infarction"[All Fields]) OR "ST elevation myocardial infarction"[All Fields] OR "stemi"[All Fields] OR "stemis"[All Fields]) OR ("non st elevated myocardial infarction"[MeSH Terms] OR ("non st"[All Fields] AND "elevated"[All Fields] AND "myocardial"[All Fields] AND "infarction"[All Fields]) OR "non st elevated myocardial infarction"[All Fields] OR "nstemi"[All Fields] OR "nstemis"[All Fields]) OR "ST elevation myocardial infarction"[All Fields] OR "ST-segment elevation myocardial infarction"[All Fields] OR "non st elevation myocardial infarction"[All Fields] OR "non st elevation myocardial infarction"[All Fields] OR "acute MI"[All Fields]))	482
Cochrane Library	(tocilizumab OR "interleukin-6 receptor antagonist" OR "interleukin-6 receptor inhibitor" OR "IL-6 receptor antagonist" OR "IL-6R antagonist" OR "anti-IL-6") AND ("myocardial infarction" OR "acute coronary syndrome" OR STEMI OR NSTEMI OR "ST elevation myocardial infarction" OR "ST-segment elevation myocardial infarction" OR "non-ST elevation myocardial infarction" OR "acute MI")	65
Embase	(tocilizumab OR "interleukin-6 receptor antagonist" OR "interleukin-6 receptor inhibitor" OR "IL-6 receptor antagonist" OR "IL-6R antagonist" OR "anti-IL-6") AND ("myocardial infarction" OR "acute coronary syndrome" OR STEMI OR NSTEMI OR "ST elevation myocardial infarction" OR "ST-segment elevation myocardial infarction" OR "non-ST elevation myocardial infarction" OR "acute MI")	1,026
Scopus	(tocilizumab OR "interleukin-6 receptor antagonist" OR "interleukin-6 receptor inhibitor" OR "IL-6 receptor antagonist" OR "IL-6R antagonist" OR "anti-IL-6") AND ("myocardial infarction" OR "acute coronary syndrome" OR STEMI OR NSTEMI OR "ST elevation myocardial infarction" OR "ST-segment elevation myocardial infarction" OR "non-ST elevation myocardial infarction" OR "acute MI")	486
